# The unveiling of a dynamic duo: hydrodynamic cavitation and cold plasma for the degradation of furosemide in wastewater

**DOI:** 10.1038/s41598-024-57038-6

**Published:** 2024-03-21

**Authors:** Federico Verdini, Anna Abramova, Luisa Boffa, Emanuela Calcio Gaudino, Giancarlo Cravotto

**Affiliations:** 1 Dipartimento di Scienza e Tecnologia del Farmaco, University of Turin, Via Giuria 9, 10125 Turin, Italy; 2 Kurnakov Institute of General and Inorganic Chemistry of the Russian Academy of Sciences, Leninsky Prospekt 31, Moscow, Russia 119991

**Keywords:** Hydrodynamic cavitation, Electrical discharge plasma, Water decontamination, Furosemide, Ecology, Chemistry, Engineering

## Abstract

The degradation in water of furosemide (FUR), a widely used diuretic drug, was herein reported. The method entails an integrated approach based on the hybridisation of hydrodynamic cavitation (HC) with electrical discharge (ED) plasma technology. This dynamic duo could increase the production of oxidising compounds in water, in particular hydroxyl radicals (OH radicals), by triggering the rapid homolytic decomposition of water molecules and avoiding the addition of external oxidants. This study clearly emphasises the effectiveness of an integrated approach to improve the degradation of pollutants in wastewater originating from active pharmaceutical ingredients (APIs). The results of HC/ED-assisted FUR degradation in the presence of radical scavengers highlight the predominant role of the radical oxidation mechanism at the gas–liquid interface of the cavitation bubble during HC/ED treatment. A comparative analysis of the three technologies—HC alone, HC/ED and UV alone—emphasised the promising potential of hybrid HC/ED as a scalable industrial technology. This is demonstrated by the higher degradation rates (100%, 10 min) when treating large volumes (5L) of wastewater contaminated with FUR (50 mg/L), even in the presence of other APIs.

## Introduction

The continuous increase in the consumption of active pharmaceutical ingredients (APIs) (human and veterinary medicines) and the often inadequate or inefficient wastewater treatment plants pose an environmental risk. The complete removal of APIs from effluents^1^ in view of municipal wastewater reuse (WWTPs)^2^ requires more efficient technologies. Among all new pharmaceuticals emerging contaminants (PEC), sulfamethoxazole (SML), acetaminophen (ACT), carbamazepine (CBZ), diclofenac (DCF), ibuprofen (IBU), caffeine (CFA), naproxen (NPX), ciprofloxacin (CPF) and furosemide (FUR) were identified as the pharmaceutical compounds with the highest occurrence in surface water, WWTPs and hospitals wastewater in concentrations ranging from ng/L to mg/L^3^. FUR is a potent loop diuretic that acts on the kidneys to ultimately increase water loss from the body, making it widely prescribed and used to treat hypertension. It has a rapid onset and short duration of action and has been used safely and effectively in both paediatric and adult patients^4^. FUR undergoes primary metabolism in the kidneys and, to a lesser degree, in the liver. Approximately 85% of the total FUR clearance is attributed to renal processes, with around 40% involving biotransformation. The major metabolites include pharmacologically active FUR glucuronide and saluamine (CSA) or 4-chloro-5-sulfamoylanthranilic acid. FUR has had many trade names including Frusemide, Uremide™, and Lasix^®^. The latter market size is expected to develop revenue and exponential market growth during the forecast period from 2023 (approximately 15 USD millions)—2030 (approximately 40 USD millions)^5^. However, FUR is categorized as one of the highest risk pharmaceutical compounds in the environment^6^. It is mostly excreted unchanged to the environment^7^ and it was detected more than the reference threshold of 100 ng/L both in inflow and effluents of WWTPs, rivers, sewage hospital^8,9^. In addition to FUR, related metabolites (saluamine and pyridinium of FUR) can be also found in raw wastewater but their removal in the WWTPs is generally very high (> 80%)^10^. However, the availability of literature data regarding the development of processes and technologies suitable for efficient drug removal from water is very limited. Only few cavitational^11^, photocatalytic^12^, and adsorption^13^ processes have been studied to efficiently remove the diuretic from water sources. Considering the ecotoxicological, mutagenic and genotoxic effects on plants, animals and humans resulting from the presence of pharmaceuticals even in low concentrations in the environment and the many limitations due to the low efficiency of wastewater treatment plants, the study and development of new efficient, sustainable and scalable technologies at industrial level is essential. Besides membrane filtration^14^, membrane bioreactor^15^, biological treatments^16^, advanced oxidation processes (AOPs) are the most promising, cost-effective and commonly used technologies for the complete degradation of persistent organic contaminants, such as FUR^11^ and other antibiotics in wastewater^17^. Considering the increasing trend in the development and application of combined or hybrid technologies for wastewater treatment in recent years^1^, AOPs have been successfully coupled with other conventional AOPs such as UV light, Fenton, ozonation^18^, ultrasound^19^, hydrodynamic cavitation^20^ and electrical discharge plasma (ED)^21–23^.

Generally, ED plasma is generated by applying a continuous or an alternate voltage between two electrodes immersed in a gas or a liquid phase to generate UV light, oxidizing compounds such as ^·^OH, ^·^H, ^·^O, H_2_O_2_, UV light, O_3_^24^ and extreme hotspot temperature (> 1000 K)^25^ for organic pollutants degradation in water^26–28^.

In view of wastewater treatment, discharge plasma can be formed:

- Over the water surface by gas ionization and excitation (N_2_, O_2_, air)^29^
- Directly in water^30^
- Inside water vapor or gas bubbles^31^

**Figure 1:**
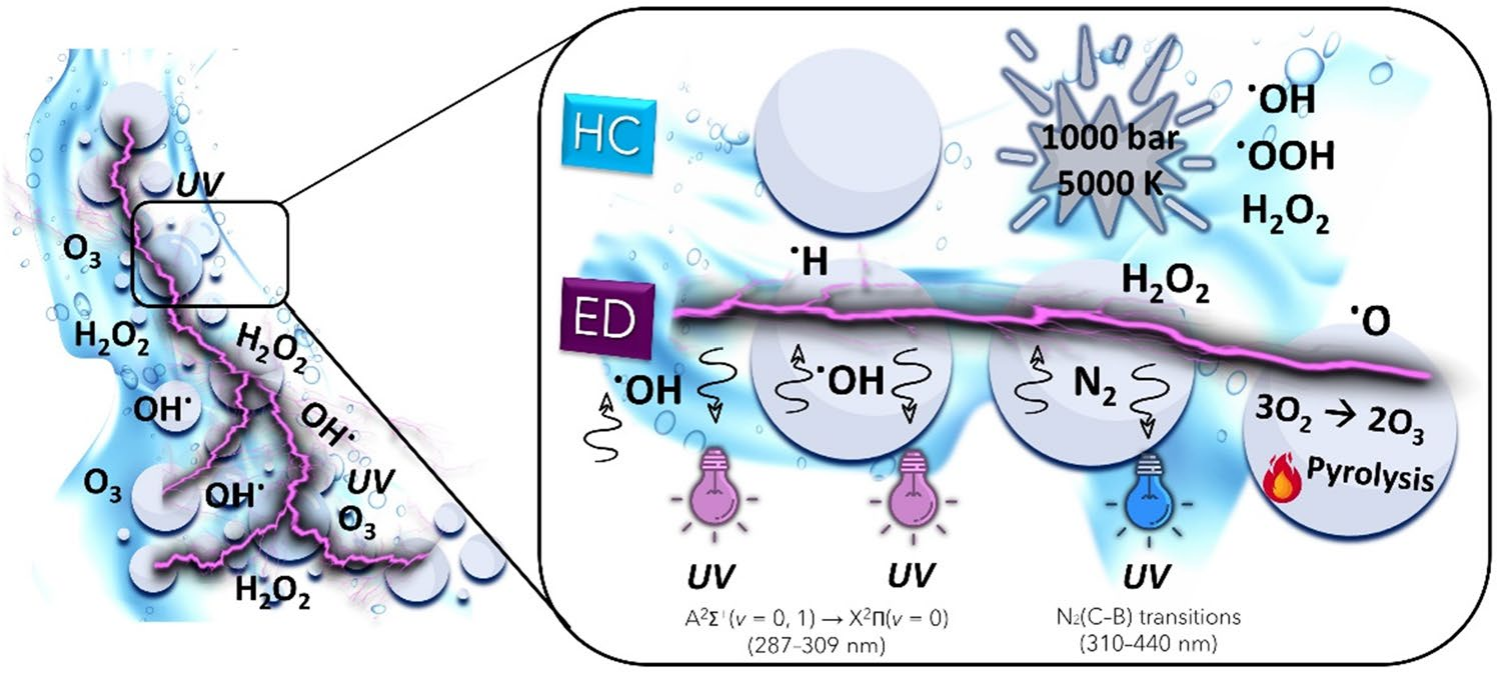
Schematic representation of the ED propagation between cavitation bubbles inside the HC/ED pilot scale reactor.

The ED plasma over the liquid surface can be generated by using one electrode placed in the gas phase above the liquid and the water phase as a counter electrode or with a second electrode immersed in the liquid. However, the efficiency of the process is influenced by the conductivity of the liquid and the gap distance. In addition, further studies should be carried out to better understand the mechanism of dissociation products of water formed in the plasma. When generating plasma directly in the liquid, numerous factors such as the type of power supply, the electrode geometry, the material properties, and the solution properties can influence the process. Furthermore, the electrical breakdown of water requires large localized electric fields of the order of megavolts per centimetre. On the contrary, large surface areas and the presence of the gas phase in gas or vapour bubbles facilitate the initiation^32^ of the discharge and subsequent propagation along the inside of the bubble at the gas–liquid interface. Usually, the temperature of the solution is kept close to the boiling point to produce bubbles containing water vapour and possibly trace gases dissolved in the liquid, while a needle and the water phase are used as electrodes. Despite experimental results demonstrating the formation of ^**·**^OH, ^**·**^H, ^**·**^O and H_2_O_2_^32^, the instrumental configuration conducive to plasma generation within bubbles requires a significant constraint on the widespread and industrial-scale utilization of this technology. In this work, we therefore present a novel approach for the application of ED plasma generated in vapour and gas bubbles in combination with a pilot-scale HC device (HC/ED reactor), which guarantees the generation of oxidising compounds (^**·**^OH, ^**·**^OOH, H_2_O_2_) from the decomposition of water molecules^33^ (due to the high energy released during the cavitation bubbles implosion). The high jet flows and the presence of cavitation bubbles at room temperature allow the propagation of electrical discharges in and between them to generate plasma, oxidizing compounds and UV light for the degradation of FUR (Fig. 1). Additional tests were carried out in the presence of radical scavengers or metronidazole (MNZ) to evaluate the efficiency of the reactor toward real wastewater treatments and to assess the main degradation mechanism between radical oxidation and pyrolysis.

## Materials and methods

### Chemicals

Furosemide (> 98%), metronidazole (> 99%), absolute ethanol, acetonitrile, methanol, and LiChroprep RP-18 silica were purchased from Sigma-Aldrich.

### Combined HC/ED reactor set-up

**Figure 2:**
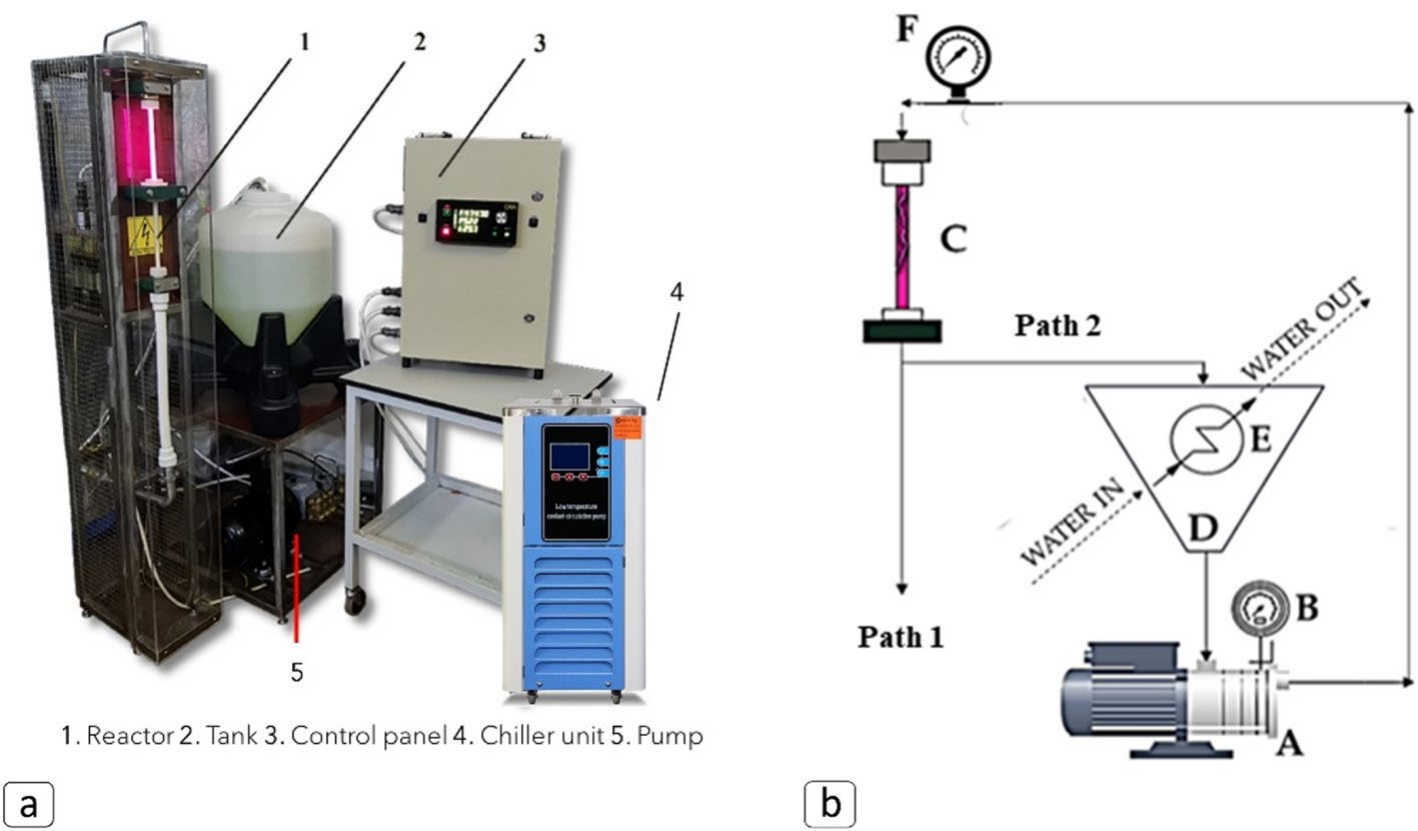
(**a**) HC/ED reactor setup; (**b**) operational setup of the HC/ED instrument in both flow-through (Path 1) and loop configuration (Path 2): (**A**) pump; (**B**) pressure gauge 1; (**C**) reaction chamber; (**D**) reservoir tank; (**E**) heat exchanger; (**F**) Pressure gauge 2.

The hybrid reactor set-up is shown in Fig. 2a. Hydrodynamic cavitation was generated using a triplex plunger high-pressure pump (3.3 kW power, SPECK Pumpen Verkaufsgesellschaft GmbH, Neunkirchen am Sand, Germany) and a 4-holed (4 mm for each hole) orifice plate placed at the top of a quartz cylinder discharge chamber (length, 200 mm; diameter, 8 mm). The inlet pressure (5–20 bar) to the orifice plate was regulated with a pressure adjustment knob on the manifold of the pump unit. Double control of the operating pressure was ensured by a pressure gauge mounted on the manifold and a second pressure gauge mounted at the top of reaction chamber. The ED plasma was generated by applying an alternating voltage (15 kV, 48 kHz, 0.6 A) between two brass electrodes positioned at the ends of the quartz cylinder (distance between the electrodes: 200 mm). A 30 L stainless steel tank equipped with an integrated coil heat exchanger was used to recirculate the wastewater. Temperature of recirculated water was kept constant at 25 ± 2 °C using a chiller unit (DLSB-5/10, Zhengzhou Keda Machinery and Instrument Equipment Co., Ltd, Zhenghou, Henan, China) linked to the heat exchanger. Samples recovered in flow mode (Path 1) can be collected directly from the output pipeline, while the samples collected in loop configuration (Path 2) can be recovered from the valve placed on the pump bypass. Additional details of the operational setup of the HC/ED instrument in both flow-through (Path 1) and loop configuration (Path 2) were reported (Fig. 2b and Fig. S1).

### General procedure for HC/ED degradation treatments of furosemide

Degradation tests were carried out on 5 L tap water (composition shown in Table S1) solutions at three different FUR concentrations (10, 30 and 50 mg/L) with an inlet pressure of 20 bar (flow rate 330 L/h) both with HC alone and combined HC/ED (48 kHz of ED frequency). The initial pH of each solution was corrected to 9 by using a 10 M solution of NaOH before each test. All experiments were carried out for a total time of 10 min and the samples were either collected in flow configuration (Path 1; Fig. 2b) and every 5 min in loop configuration (Path 2; Fig. 2b). Temperature of recirculated water was kept constant at 25 ± 3°C. Based on the volume of the reaction chamber (10 mL), the residence time for treatment performed at 5500 mL/min (330 L/h) in flow mode was 0.0018 min. For 10 min loop experiments, the actual residence time was 0.02 min, according to the equations reported in the supporting information (Equation S1-3).

### General procedure for UV and UV/H2O2 degradation treatments of furosemide

FUR was subjected to degradation treatments in a batch configuration by using a UV lamp (Kessil PR160L, 390nm) characterized by an average emission intensity of 137 mW/cm^2^ on an 8 cm^2^ exposed surface at 6 cm. Each treatment was performed on a 200 mL FUR solution of 10 mg/L at pH = 9 in a borosilicate glass beaker (internal diameter: 65 mm; height of the treated liquid: 65 mm) coated with aluminium foil to avoid UV radiation loss, under magnetic stirring at 300 rpm, and irradiated from the surface exposed to air placed at 6 cm from the UV lamp as shown in Figs. S2 and S3. A ventilation system ensured both the UV lamp and reacting solution cooling, which was kept constant at a temperature of 25 °C. The system was placed in a dark aspiration hood to avoid external irradiation influence. The same set-up was exploited for the combined UV/H_2_O_2_ treatments performed with a FUR and H_2_O_2_ 1:100 molar ratio. Both UV and combined UV/H_2_O_2_ batch procedures were conducted for 5 and 10 min. A qualitative test was also performed for a total treatment time of 3 h under UV irradiation only.

### Concentration of treated furosemide samples

Samples collected during the degradation processes were concentrated by using a manually packed column prepared with 5 g of LiChroprep RP-18 silica with particle sizes in the range of 40–63 µm (Merck Millipore, Burlington, USA). In detail, 100 mL of each collected sample were eluted under vacuum through the column and the analytes were recovered with 16 mL of methanol, with a consequent concentration factor value of 6.25. The recovered organic fraction was subsequently injected into the HPLC system.

### Analysis

The quantitative analysis of treated samples was achieved through a Waters (Waters Corp., Milford, USA) high performance liquid chromatograph equipped with a 2998 photodiode array (PDA) detector (UV/DAD, Waters Corp., Milford, USA). The best selected wavelength for FUR detection was 235 nm. The best selected wavelength for MNZ detection was 318 nm. The chromatographic column used was a reversed phase 4.6 mm × 150 mm Kinetex C18 (Phenomenex) with 5 µm particles. The column and the HPLC system were kept in ambient conditions. The mobile phase was acetonitrile/HCOOH (0.1%)–water/HCOOH (0.1%) and a gradient mode was used (Supporting information) at a flow rate of 1.0 mL/min. The injection volume was 20 µL.

### Preparation of calibration curve

A stock solution (2 mg/mL) was prepared by dissolving 20 mg of FUR in 10 mL of methanol. Standard solutions (0.50, 0.25, 0.10, 0.075, 0.050, 0.025, 0.010, 0.005 mg/mL) were prepared by suitable dilutions in 2 mL volumetric flasks. Calibration curve is reported in Fig. S4. In addition, a standard 10 mg/L solution of metronidazole (MNZ) was prepared and subsequently concentrated by using LiChroprep RP-18 silica as described previously. The concentrated solution was injected into HPLC, and the resulting peak area was used as a starting reference to calculate the degradation rate.

### Kinetic modelling

The kinetic evaluation of the HC/ED treatments was conducted by fitting the experimental data to pseudo-first-order kinetic model (Eq. 1). Linear regressions for HC/ED experiments are reported in Fig. S5. The calculated kinetic constant values are listed in Table S3.

1 $$\frac{d\left[ A \right]}{{dt}} = - k\cdot\left[ A \right]$$

## Results

### Influence of furosemide initial concentration on HC/ED degradation tests

**Figure 3:**
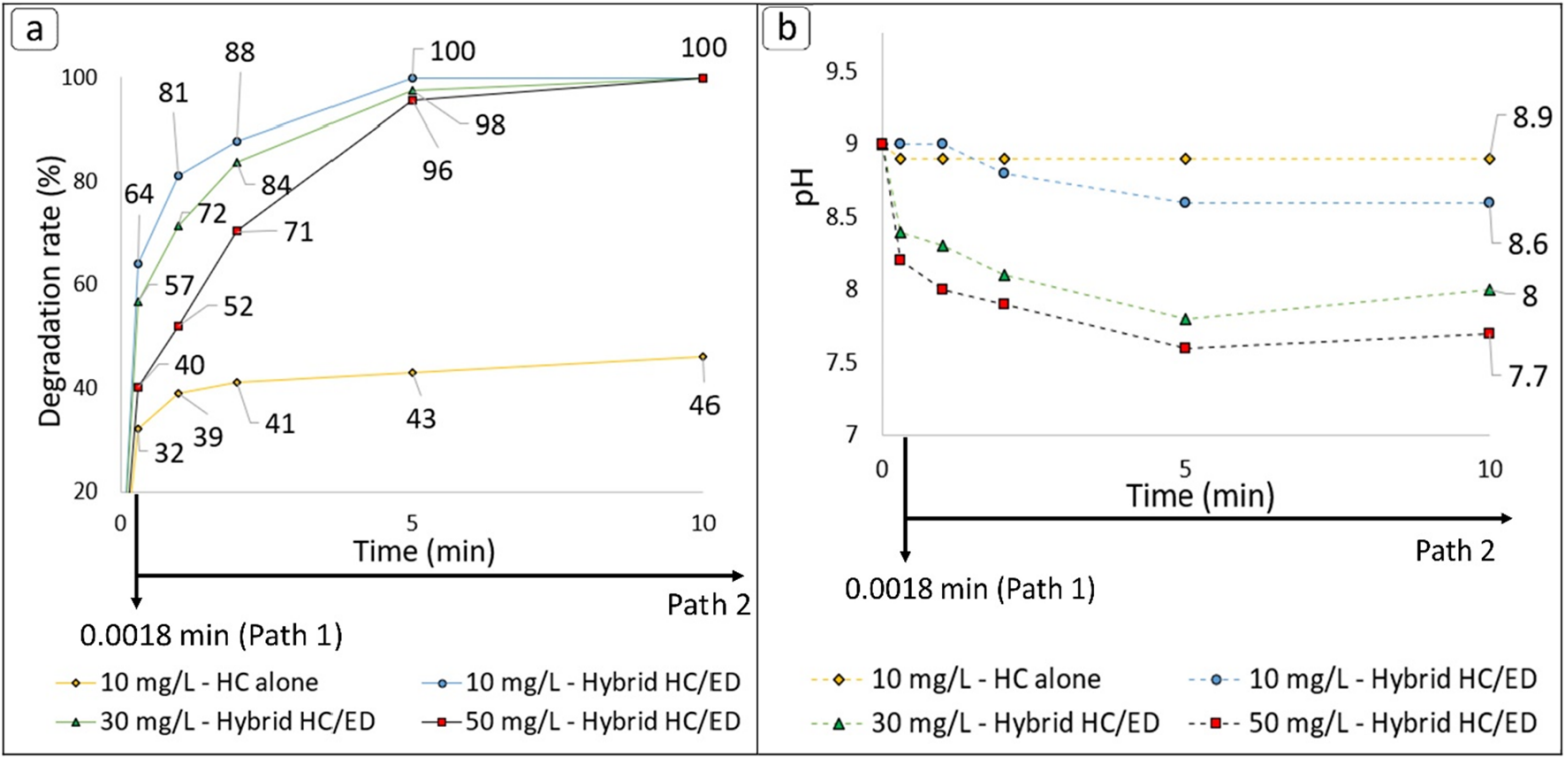
Results of hybrid HC/ED treatments. (**a**) Degradation rate values. (**b**) pH variation during treatments.

To investigate the efficiency of the prototype reactor in the degradation of various concentrations of FUR, the first set of experiment were conducted with different initial concentrations (c_0_) of analyte (10, 30 and 50 mg/L) working at 20 bar of inlet pressure and 48 kHz of ED plasma frequency, both in flow and loop configurations (Fig. 3). Considering the high drug concentration, the pH of tap water (7.6) was corrected to 9 with a 10 M solution of NaOH to facilitate the FUR solubility. The pH variation during the treatments was also measured. In respect to the test performed with a starting FUR concentration of 10 mg/L, a complete degradation was observed after only 5 min of treatment in loop configuration, while the flow mode allowed to observe a degradation rate of 64% (final FUR concentration: 3.6 mg/L). The kinetic constant for this treatment was 0.8294 min^−1^. For the sake of comparison, the same amount of FUR was also subjected to a degradation treatment under the effect of HC alone, without the contribute of ED plasma. During the non-hybrid treatment (HC alone), a 32% of degradation rate (final FUR concentration: 6.8 mg/L was observed in flow mode and a slight increase up to 43% and 46% (final FUR concentrations: 5.7 and 5.4 mg/L, respectively) was observed after 5 and 10 min of treatment, respectively. Unfortunately, no kinetic models were found for the HC alone test. The flow mode and 5 min loop treatment allowed to obtain, respectively, a 57% and 98% degradation rate values (final FUR concentrations: 13.0 and 0.7 mg/L, respectively) during the treatment conducted on the 30 mg/L FUR solution, demonstrating the same reactor efficiency observed during the previous tests. The kinetic constant slightly decreased to 0.6763 min^−1^ from that observed in the previous test (0.8294 min^−1^). The increase of initial concentration up to 50 mg/L slightly affected the degradation in flow (40%, final FUR concentration: 18.0 mg/L) but a near quantitative degradation of 96% (final FUR concentration: 1.3 mg/L) was observed again after 5 min of treatment. For this experiment the kinetic constant was 0.6452 min^−1^. Overall, a treatment time of 10 min ensured a complete degradation of FUR for each starting concentration value, even though the kinetic constant value decreased with increasing the starting FUR concentration. In respect to the monitored pH during treatments (Fig. 3b), it was overall observed that as the initial concentration of FUR increased, the pH gradually decreased as a direct result of the greater amount of substrate available to be converted to organic acids under the extreme oxidative environment generated by hybrid HC/ED.

The observed results suggested that the pilot scale hybrid reactor maintained its efficiency even at very high starting FUR concentration values, which were chosen specifically to demonstrate the capabilities of the hybrid pilot scale reactor, even under the flow mode. The showed results demonstrate the actual chance of using the hybrid HC/ED technology to treat FUR-polluted water at industrial level in flow, considering that the FUR concentrations generally observed in hospital effluent or rivers/streams are in the range of µg/L^11^, which is significantly lower than that used in the previously described experiments. However, it should be considered that the treatment of a real effluent could be affected or limited by the presence of additional contaminants or substances that can quench the radical oxidation.

### Influence of scavenger addition on HC/ED degradation tests of furosemide

To further test the efficiency of the HC/ED pilot scale reactor in presence of ^**·**^OH scavengers or other pollutants, and to investigate the possible contribution of pyrolysis reactions in the degradation of contaminants, different degradation treatments were performed in presence of alcohols or an additional drug. As already demonstrated in a previous works^24–26^, the concentration of oxidizing compounds (UV light, H_2_O_2_, O_3_ and ^**·**^OH) inside the recirculating wastewater can be enhanced by to the combination of the ED plasma with HC. Nevertheless, alcohols such as ethanol (EtOH) can scavenge ^**·**^OH^34^ Eq. (2) and a competition between the ED plasma-generated radicals and organic contaminants can take place, limiting their degradation rates.

2 $${\text{CH}}_{3} {\text{CH}}_{2} {\text{OH}} + \cdot {\text{OH}} \to \cdot {\text{CH}}_{2} {\text{CH}}_{2} {\text{OH}} + {\text{H}}_{2} {\text{O}}$$

**Figure 4:**
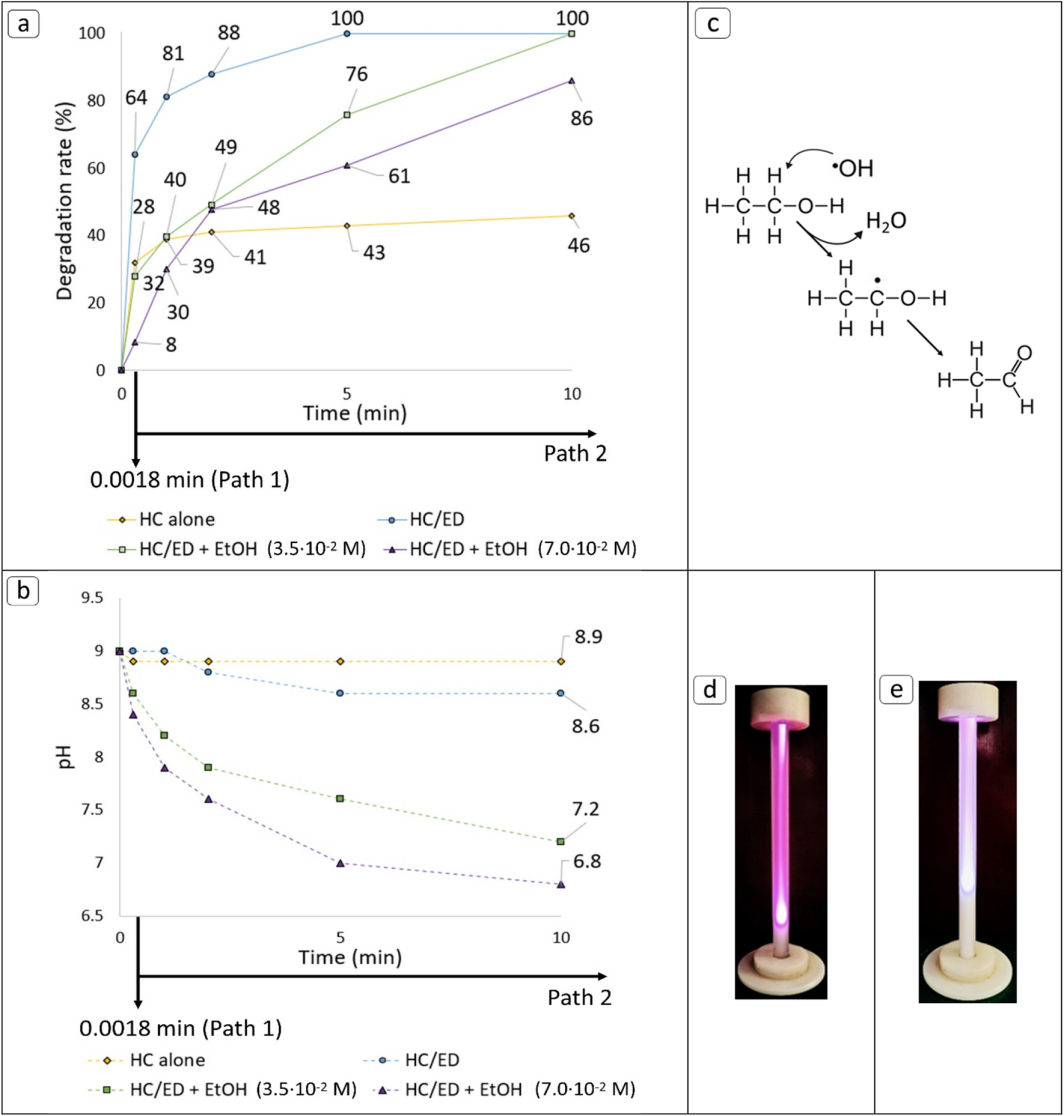
(**a**) Results of hybrid HC/ED treatments performed in presence of EtOH. (**b**) pH variation during treatments. (**c**) Scheme of the radical oxidation of EtOH. (**d**) Benchmark ED plasma colour. (**e**) Quenched ED plasma colour during treatment performed in presence of EtOH.

The relatively high EtOH volatility (vapor pressure = 55 mmHg) could facilitate its diffusion into cavitation bubbles during their generation, extinguishing the oxidative radical reactions that occurs at the gas(bubble)-liquid (bulk solution) interface. However, since there is no experimental evidence of EtOH diffusion into cavitation bubble, a 10 mg/L FUR solution was treated in presence of EtOH (3.5 × 10^–2^ M and 7.0 × 10^–2^ M) with an inlet pressure of 20 bar and an overall decrease in the degradation rate was observed for both flow and 5 min treatments (Fig. 4a).

**Figure 5:**
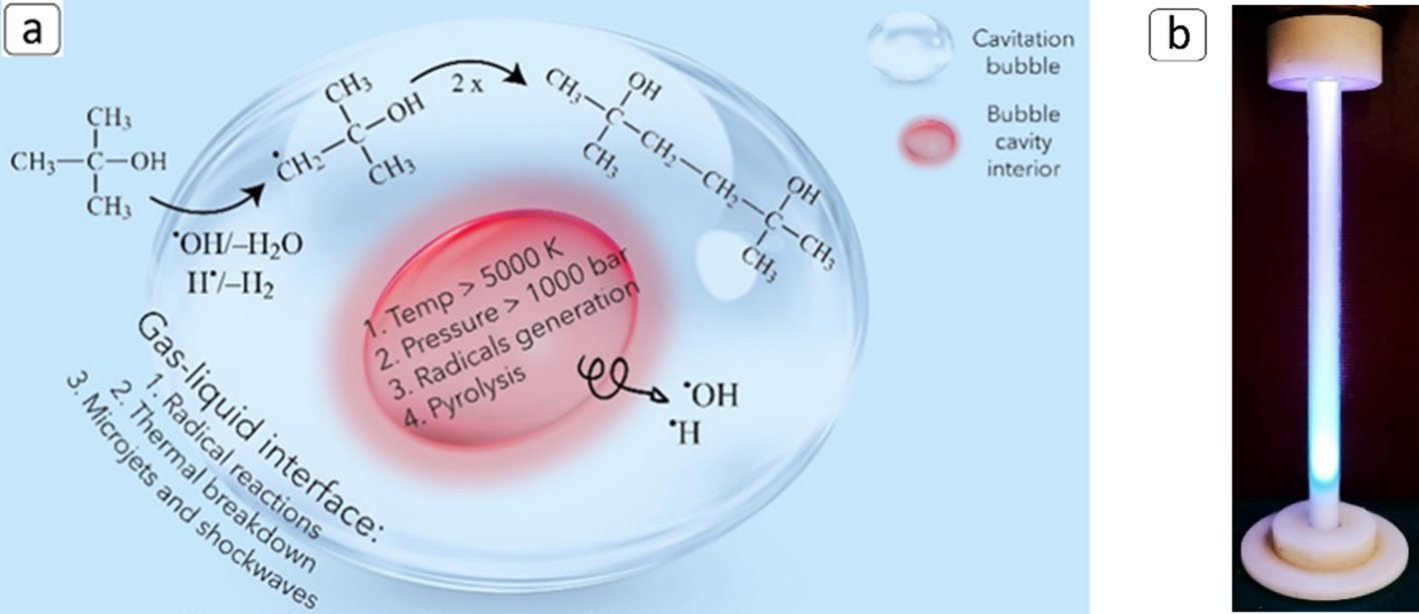
(**a**) Schematic representation of *t*-BuOH quenching activity in the gas–liquid interface of the cavitation bubble. (**b**) ED plasma colour in presence of *t*-BuOH.

In detail, during the treatment performed with a 3.5 × 10^–2^ M concentration of EtOH the degradation rate decreased by a value of 56% in flow mode (final FUR concentration: 7.2 mg/L) and a value of 24% (final FUR concentration: 2.4 mg/L) after 5 min, while the complete degradation was observed after 10 min of treatments, with a resulting kinetic constant value of 0.2584 min^–1^. A scavenger concentration of 7.0 × 10^–2^ M caused degradation rate reductions by values of 88% and 39% for the flow and 5 min treatments (final FUR concentrations: 9.2 and 3.9 mg/L), respectively, with an 86% degradation rate after 10 min (final FUR concentration: 1.4 mg/L). As expected, the kinetic constant of the latter test (0.1870 min^–1^) was lower than the previous experiment (0.2584 min^–1^) However, despite the presence of scavengers, 5 min of treatment were sufficient to achieve higher degradation than observed in cavitation alone (43%). The kinetic modelling confirmed the scavenger effect explicated by EtOH. In respect to the pH variation of the water matrix, during the quenched experiments conducted with EtOH concentration values of 3.5 × 10^–2^ M and 7.0 × 10^–2^ M, a pH decreases to 7.2 and 6.8, respectively, was documented (Fig. 4b). On the contrary, during the unquenched test the minimum reached pH value was 8.6. The significant decrease in pH may be ascribe to the formation of acetic acid derived from a partial oxidation of EtOH via a two-step reaction: a first radical EtOH oxidation towards acetaldehyde by ^**·**^OH attack ^35^ (Fig. 4c) followed by a further oxidation step of acetaldehyde supported by the extreme oxidant HC/ED environment to generate acetic acid. Two additional treatments were performed on tap water alone (pH = 9) in presence of EtOH and the final pH values after 10 min were 7.8 and 7.4 with starting EtOH concentrations of 3.5 × 10^–2^ M and 7.0 × 10^–2^ M, respectively. These results, in combination with an observed constant pH during a further test performed with only tap water (pH = 9), could confirm the contribute of EtOH oxidation previously hypothesized. The results of the experiments showed that EtOH acted as a ^·^OH quencher during the treatments, limiting the radical oxidation mechanism. In addition, during the described treatments, a switch of ED plasma colour from the benchmark pink-purple (Fig. 4d) to a pale pink-blue-white (Fig. 4e) was observed. Under unperturbed conditions, both the HC and HC/ED plasma-generated ^**·**^OH reach their excited state due to the energy provided by the electrical discharge (ED). Their subsequent relaxation determines the emission of UV light with a wavelength in the range of 287–309 nm (A^2^Σ^+^(v = 0, 1) → X^2^Π(v = 0))^36^, justifying the bright pink-purple plasma colour. In presence of EtOH, the decrease of ^**·**^OH concentration also determines a decrease in the UV light emission, with a reduction of an additional source of oxidation. Despite the presence of EtOH, a partial degradation of the analyte has nevertheless been observed, probably due to the presence of HC/ED-generated H_2_O_2_ and O_3_ or, possibly, to the contribute of pyrolysis reaction which can occur during cavitational treatments^37,38^ in the core of cavitation bubbles (Fig. 5a). To better underline a plausible contribution of pyrolysis reaction in the FUR degradation, additional tests were carried out in presence of *tert*-butyl alcohol (*t*-BuOH), as it has been demonstrated that it can diffuse into the cavitation bubbles during their generation and subsequent growth due to its volatility (vapor pressure = 46 mmHg). This phenomenon allows *t*-BuOH to exert its quenching effect, acting as a ^·^OH scavenger both in the gas phase and in the gas–liquid interface of the cavitation bubble^39,40^ as shown in Fig. 5a. Considering the colour variation of the emitted light observed during the cavitational treatments in presence of EtOH (Fig. 4d and 4e), a preliminary test was conducted in water with *t*-BuOH (3.5 × 10^–2^ M) to investigate possible differences in the quenching activities of the two alcohols. In contrast of what observed in presence of EtOH, the addition of *t*-BuOH determined a variation of the benchmark light towards a more intense blue-white light (Fig. 5b). The extensive decrease of pink light could be due to the higher rate constant reaction of *t*-BuOH with ^·^OH (1.08 × 10^12^ cm^3^ molecule^–1^ s^–1^)^41^ than that of EtOH (0.4 × 10^12^ cm^3^ molecule^–1^ s^–1^)^42^, probably leading to a greater contribute of both the H^β^ radical emitted light at 490 nm (blue region of the visible spectrum), determined in a previous work^23^, and typical N_2_ emission between 400 and 440 nm (N_2_(C–B) transitions) observed during the generation of glow plasma in presence of water^26^. However, the complete understanding of the physicochemical phenomena that occurred during the described tests is difficult and requires further specific investigations.

**Figure 6:**
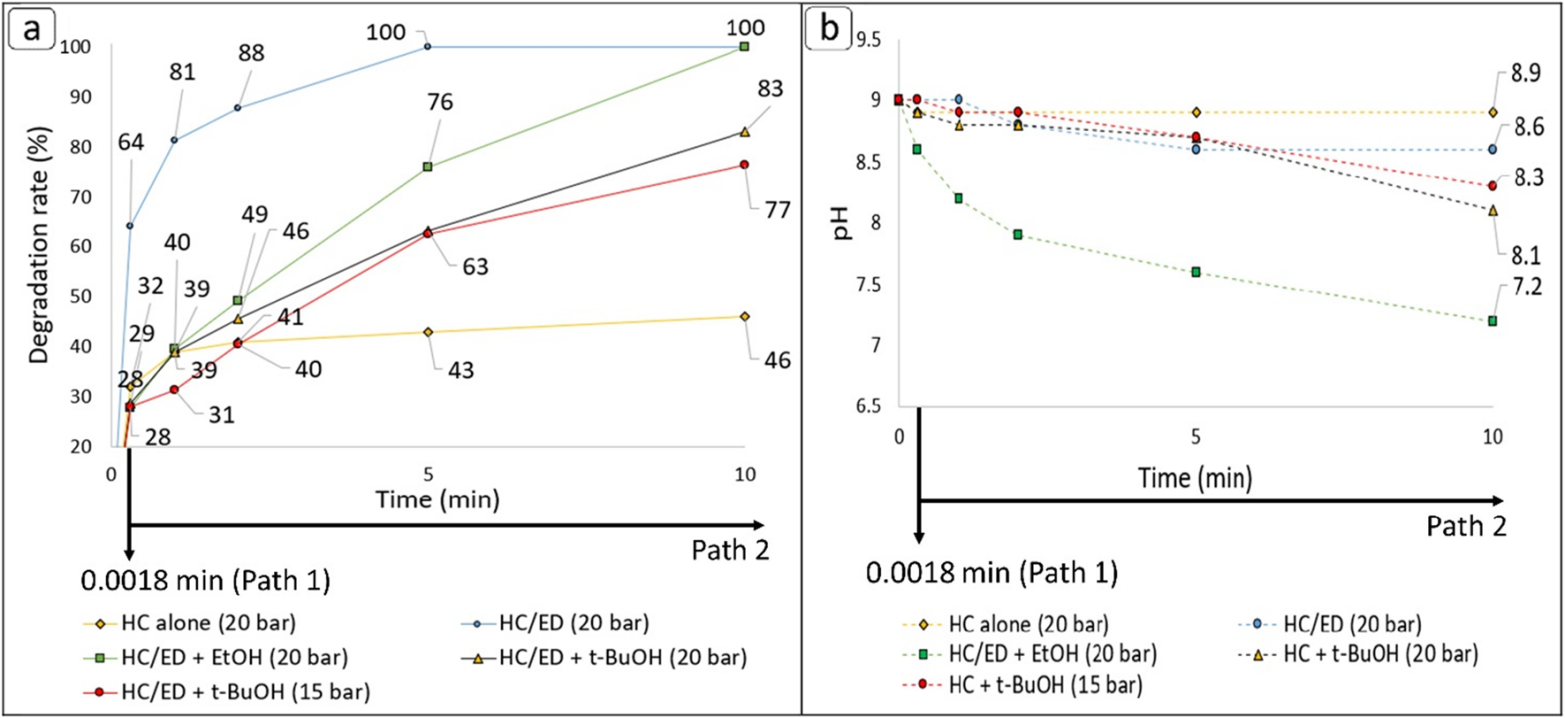
(**a**) Results of HC/ED treatments performed in presence of *t*-BuOH. (**b**) pH variation during the treatments.

Therefore, a degradation test on a 10 mg/L FUR solution was performed in the presence of *t*-BuOH (3.5 × 10^–2^ M) at 20 bar. Overall, a decrease of FUR degradation rate was observed compared to the treatments in presence of EtOH. In detail, the degradation rate with *t*-BuOH in flow mode (29%, final FUR concentration: 7.1 mg/L) was like that observed in presence of EtOH (28%), but a 17% decrease in the extent of degradation was observed for both 5 and 10 min of loop treatment (final FUR concentrations: 3.7 and 1.7 mg/L), respectively, in respect to the test performed with EtOH (Fig. 6a). The kinetic constant of such test was 0.1581 min^–1^, which was lower than that observed in presence of EtOH. Despite the results could demonstrate the higher efficiency of *t*-BuOH in the ^**·**^OH radicals quenching than EtOH, foaming was observed inside the reaction chamber after 1 min of loop treatment (Fig. S6), probably due to the microscopic phase separation at cluster level (i.e. coexistence of water-rich clusters and organic cosolvent-rich clusters) generated during acoustic cavitational treatments carried out in presence of a binary water-organic solvent mixture^43^. Under acoustic cavitation, water molecules promote self-association of organic molecules as a balance of interactions controlling the microscopic structure in the solution. The presence of foam caused a reduction of the cavitation intensity (reduction of cavitation plume length along the reaction chamber) and the consequent reduction of the ED plasma extension as a direct consequence, contrary to what observed during the flow mode treatment and the preliminary test with *t*-BuOH previously described (Fig. 5b). Due to this, to confirm the faster reaction between* t*-BuOH and ^**·**^OH (*k* = 1.08 × 10^12^ cm^3^ molecule^−1^ s^−1^) than EtOH (0.4 × 10^12^ cm^3^ molecule^−1^ s^−1^), an additional treatment was performed lowering the inlet pressure to 15 bar to reduce foaming and to avoid possible misleading in the interpretation of the data. The results obtained at 15 bars (k = 0.1314 min^−1^) were not significantly different to the degradation rates observed at 20 bar (Fig. 6a), confirming both the contribute of only *t*-BuOH scavenging (and not to foaming) in decreasing degradation efficiency at 20 bar, and the faster *t*-BuOH quenching reactivity than EtOH. In respect to the final pH values reached at the end of the performed treatments (Fig. 6b), in presence of *t-*BuOH were observed slight pH decreases (8.1 at 20 bar and 8.3 at 15 bar) with respect to the unquenched treatment (8.6). However, the addition of* t*-BuOH may lead to the formation of different by-products compared to benchmark experiment, thereby explaining the slight further decrease in pH. In addition, considering that *t*-BuOH is resistant to oxidation due to its tertiary alcohol structure, the conclusive pH values from tests conducted with this scavenger did not exhibit a significant decrease, in contrast to the observed trend in the presence of EtOH.

Overall, the results obtained in the presence of radical scavengers show that the mechanism of radical oxidation at the interface between gas and liquid cavitation bubbles during HC/ED treatments is the major contributor. However, considering the high degradation rate observed despite the presence of EtOH and *t*-BuOH, a possible contribution of the pyrolysis reaction cannot be excluded. In this regard, further studies should be conducted in the future to gain a deeper understanding of the hybrid HC/ED innovative technology.

**Figure 7:**
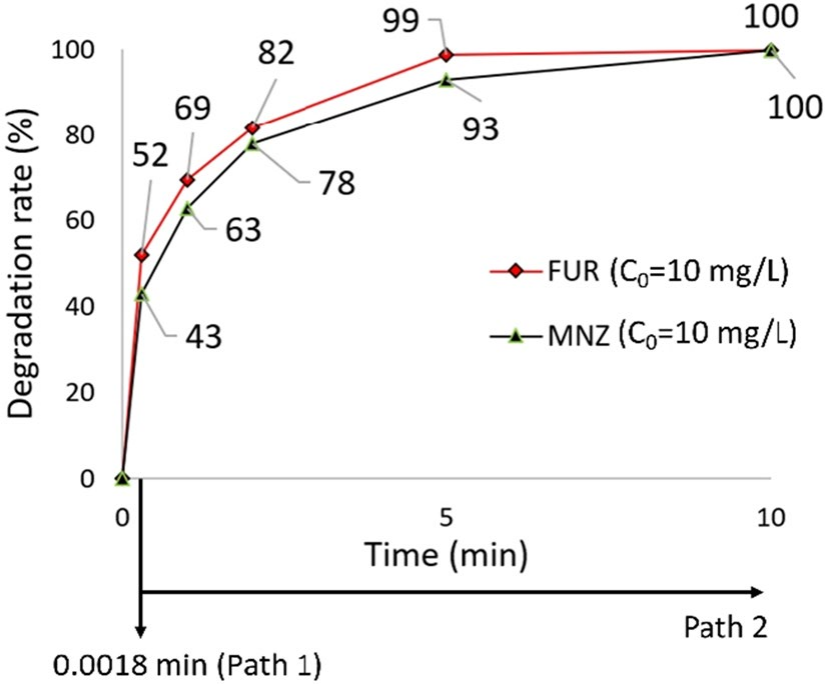
Results of simultaneous treatment of FUR and MNZ mixture under HC/ED treatment.

In view of a real industrial application of the hybrid HC/ED technology, a further experiment was conducted also in presence of another antibiotic (the metronidazole (MNZ)) to assess the efficiency of the treatment in the simultaneous degradation of two different pollutant APIs. For this purpose, a 5 L solution with a total drug loading of 20 mg/L (10 mg/L of FUR + 10 mg/L of MNZ) was treated at 20 bars for a maximum treatment time of 10 min (Fig. 7). The treatment performed in flow mode allowed to observe a FUR and a MNZ degradation rates of 52% and 43%, respectively, while the 5 min loop test guaranteed a quantitative FUR degradation (99%) and a 93% MNZ abatement. After 10 min both the diuretic (FUR) and the antibiotic (MNZ) were completely degraded, demonstrating the maintenance of the HC/ED reactor efficiency even in the case of treatments conducted in the presence of two different contaminants for prolonged treatment time. However, the addition of MNZ slightly affected the FUR abatement during the flow experiment from a degradation rate; indeed, the FUR degradation rate decreased from 64%, obtained in the treatment without MNZ, to 52%. On the contrary, from a kinetic point of view, the kinetic constant of FUR degradation in presence of MNZ 0.8413 min^−1^ was higher than that of FUR alone (0.8294 min^−1^).

Anyway, considering the very high initial drugs loading (20 mg/L) which deviates significantly from the actual concentrations of the two drugs generally found in several effluents (µg/L), the slight decrease in FUR abatement observed during the flow treatment can be considered insignificant in the case of treatments performed on real drug-polluted wastewater. In addition to the scavenging effect exerted by organic substances such as alcohols or other drug pollutants, certain inorganic substances commonly present in wastewater and/or in drinking water can also quench ^**·**^OH. Among the common inorganic ions present in tap water (e.g. Ca^2+^, Mg^2+^, Cl^−^, SO_4_^2−^, HCO_3_^−^ etc.), variation of bicarbonate concentration could induce scavenging effect because of the competitive reactions of carbonate, bicarbonate, and FUR with ^·^OH (Eqs. 3 and 4)^31^.

3 $${\text{CO}}_{3}^{2 - } + \cdot {\text{OH}} \to \cdot {\text{CO}}_{3}^{ - } + {\text{OH}}^{ - }$$

4 $${\text{HCO}}_{3}^{ - } + \cdot {\text{OH}} \to \cdot {\text{CO}}_{3}^{ - } + {\text{H}}_{2} {\text{O}}$$

Considering the variable average concentration of HCO_3_^−^ in the range of 200–500 mg/L observed in tap waters analysed in different Italian cities (Table S2), a degradation treatment was performed with the addition of bicarbonate to investigate the reproducibility of the degradation process as a function of geographic water composition in case of drinking water treatments. The starting HCO_3_^−^ concentration of the available tap water (257 mg/L Turin, Italy) was increased up to 402 mg/L (2.4 × 10^–3^ M) but no scavenger effect has been observed during the treatments due to both low HCO_3_^−^ concentration and low-rate constant (8.5 × 10^9^ cm^3^ mol^−1^s^−1^)^44^ of the reaction of bicarbonate ion with hydroxyl radicals.

### UV, H_2_O_2_ and UV/H_2_O_2_ degradation tests for furosemide

**Figure 8:**
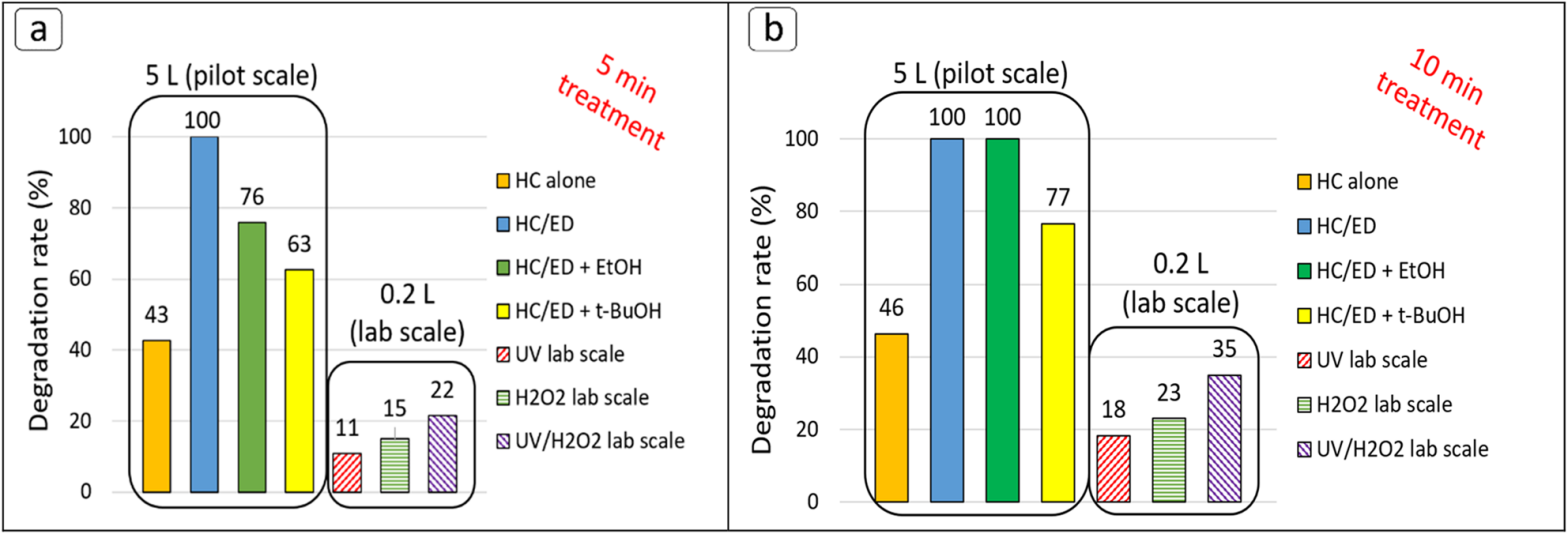
Results of UV and UV/H_2_O_2_ treatments and comparison with HC/ED performance. (**a**) Results obtained after 5 min (**b**) Results obtained after 10 min.

To compare the efficiency of the pilot scale HC/ED prototype reactor with other AOPs, additional lab-scale batch treatments were performed on a 0.2 L solution of FUR with a starting concentration of 10 mg/L and a pH value of 9, under UV irradiation alone (390 nm, average intensity of 137 mW/cm^2^) or in presence of H_2_O_2_ and under combined UV/H_2_O_2_ (1:100 FUR:H_2_O_2_ molar ratio). A preliminary prolonged degradation test of 3 h was conducted under UV irradiation only, which allowed to reduce the starting antibiotic concentration by a value of 88%. Moreover, in contrast to what observed during the HC/ED treatments (Fig. S7), the HPLC analysis clearly revealed the formation of a by-products (Fig. S8) during the UV degradation test, probably due to the different degradation pathways of UV and HC/ED treatments or to the incapability of UV light in the degradation of these compounds. Subsequently, UV-irradiated solutions were treated for 5 and 10 min to directly compare the results obtained with the pilot scale HC/ED reactor. Those experiments revealed a FUR degradation rate of 11% and 18% after 5 and 10 min of treatment time, respectively, confirming the suffering of FUR to photochemical degradation^45,46^. The addition of H_2_O_2_ allowed an increase of the UV alone degradation rates up to 22% and 35% due to the additional ^•^OH radicals generated by the UV-induced homolytic bond cleavage of H_2_O_2_. As shown in Fig. 8, the degradation rates observed for UV, H_2_O_2_ and combined UV/H_2_O_2_ lab-scale treatments conducted for 5 and 10 min were lower than the analogous treatments (HC, HC/ED, HC/ED + EtOH and HC/ED + *t*-BuOH) performed at pilot scale, overall. In addition, further UV and UV/H_2_O_2_ treatments were tried on a 0.5 L FUR solution with a starting concentration of 10 mg/L for a total time of 5 min. The 2.5-fold scale up of lab scale treatments exceeded the effective operating limit, as only a 1% degradation was observed in both the case of UV alone and in the combined process with H_2_O_2_. In detail, the lab-scale findings demonstrated even lower treatment efficiency compared to the pilot-scale operation with the use of HC as the sole treatment agent (43% of degradation rate), indicating the reactor's remarkable effectiveness even when employing a non-hybrid approach. The comparison of the three technologies revealed how the hybrid HC/ED can be considered a promising technology for its scalability at industrial level considering the higher degradation rates in treating large volumes of polluted wastewater, even in a non-hybrid approach (HC) and in presence of radical scavenger compounds.

### Energy efficiency estimations of HC/ED-assisted furosemide degradation tests

Aiming to compare the energy efficiency of the novel HC/ED hybrid technology (used in this work) with other existing non-thermal plasma treatment technologies, an estimation of the HC/ED energy efficiency was made based on the reactor energy consumption (displayed on the instrument control panel). For the hybrid HC/ED treatments performed with different furosemide output concentrations, the highest energy efficiency (350 mg/kWh) was recorded for the degradation test performed with a 50 mg/L FUR solution. The energy efficiency decreased to 226 mg/kWh and 82 mg/kWh during the treatment performed with initial FUR concentrations of 30 and 10 mg/L, respectively. In the quenched test, the highest efficiency was recorded during the experiment in the presence of a 3.5 × 10^–2^ M EtOH solution (74 mg/kWh) and decreased to 62 mg/kWh with increasing EtOH concentration (7.0 × 10^–2^ M), and to 58 and 55 mg/kWh in the presence of *t*-BuOH (3.5 × 10^–2^ M) at 20 and 15 bar inlet pressure, respectively. For the sake of comparison, Hu et al*.*^47^ reported an energy efficiency of 7 mg/kWh for the degradation of a ciprofloxacin antibiotic (C_0_ = 25 mg/L) with a value of 84.1% for a 24-min plasma jet DBD treatment reactor, while Guo et al*.*^48^ reported an energy efficiency of 67.3 mg/kWh for the degradation (76%) of enrofloxacin (C_0_ = 20 mg/L). In general, the energy efficiencies of the hybrid HC/ED reactor are comparable (for an initial concentration of 10 mg/L FUR) or even higher (for FUR C_0_ of 30 and 50 mg/L) than the values reported in the literature for other non-thermal plasma systems^49^. All calculated energy efficiency values are listed in Table S3.

## Conclusion

The present study unveils a breakthrough: the successful integration of cold plasma technology with cavitation processes for the efficient degradation of FUR (50 mg/L) within considerable volumes (5L) of circulating water (330 L/min). The highest energy efficiency (350 mg/kWh) was registered for the degradation test performed with a FUR solution of 50 mg/L. Remarkably short treatment times (i.e., 10 min or less) were demonstrated. The superior advantages of this HC/ED cold plasma hybrid technology compared to hydrodynamic cavitation alone or UV–Vis methods were clearly demonstrated. Quantitative degradation of FUR, either in isolation or in combination with another active pharmaceutical ingredient (metronidazole), was achieved in 10 min, even in the presence of radical scavengers (i.e. ethanol). This emphasises the significant contribution of the radical oxidation mechanism at the interface between gas and liquid cavitation bubbles during HC/ED treatments. These findings pave the way for the potential industrial application of HC/ED cold plasma technology in the treatment of pharmaceutical wastewater.

### Supplementary Information

Supplementary Information.

## Data Availability

The authors declare that the data supporting the findings of this study are available within the paper and its Supplementary Information files. The datasets generated and/or analysed during the current study are available in the GOOGLE DRIVE repository, source data are provided with this paperlink for raw data: https://drive.google.com/drive/folders/1mEAnWpSEAPHibu7FIWW0M_yePx5gZV6C?usp=sharing
