# Supplementary material for: The unveiling of a dynamic duo: hydrodynamic cavitation and cold plasma for the degradation of furosemide in wastewater
Source: Sci Rep. 2024 Mar 21;14:6805. doi: 10.1038/s41598-024-57038-6 (PMC10957998; doi:10.1038/s41598-024-57038-6)
Supplement: Supplementary file 1 — Supplementary Information. [file 41598_2024_57038_MOESM1_ESM.docx]

**SUPPORTING INFORMATION**

**The unveiling of a dynamic duo: hydrodynamic cavitation and cold plasma for the degradation of furosemide in wastewater**

Federico Verdini,^1^ Anna Abramova,^2^ Luisa Boffa,^1^ Emanuela Calcio Gaudino,^1^* Giancarlo Cravotto^1^*

^1^ Dipartimento di Scienza e Tecnologia del Farmaco, University of Turin, Via Giuria 9, 10125 Turin (Italy).

^2^ Kurnakov Institute of General and Inorganic Chemistry of the Russian Academy of Sciences, Leninsky Prospekt 31, 119991, Moscow (Russia).


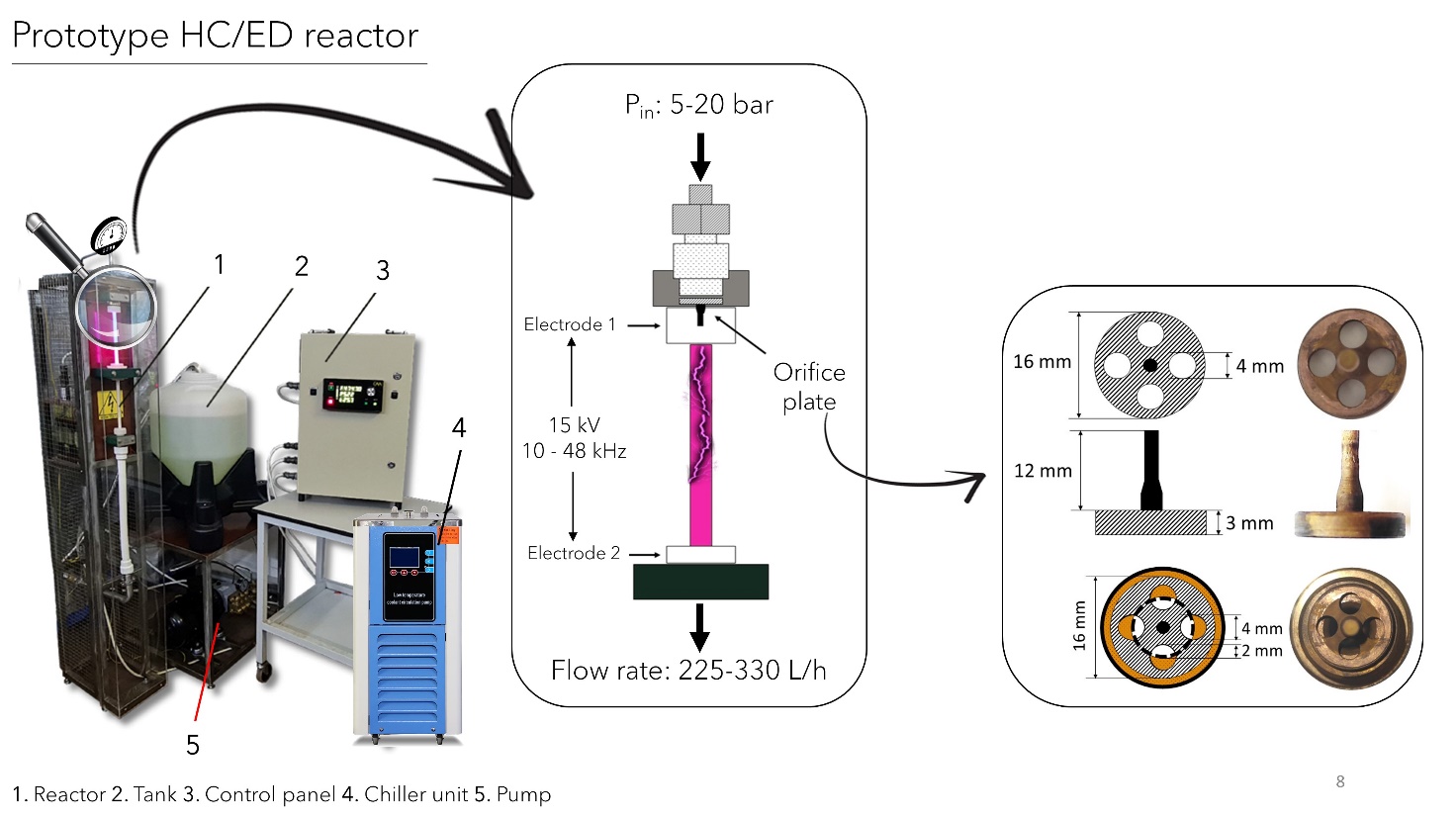


**Figure S1.** Details of the HC/ED reaction chamber

| **Parameter** | **[H^+^]** | **Dry residue (180°C)** | **Total hardness** | **Conductivity** | **Ca^2+^** | **Mg^2+^** | **NH_4_^+^** | **Cl^-^** | **SO_4_^-^** |
| --- | --- | --- | --- | --- | --- | --- | --- | --- | --- |
| Measurement unit | pH | mg/L | °F | µS/cm | mg/L | mg/L | mg/L | mg/L | mg/L |
| Turin (District 8) | 7.7 | 369 | 26 | 509 | 76 | 16 | < 0.05 | 23 | 48 |
|  | **K^+^** | **Na^+^** | **NO_3_^-^** | **HCO_3_^-^** |  |  |  |  |  |
|  | mg/L | mg/L | mg/L | mg/L |  |  |  |  |  |
|  | 2 | 13 | 16 | 257 |  |  |  |  |  |

**Table S1.** Tap water composition. Analysis provided by SMAT srl (Società Metropolitana Acque Torino). <https://www.smatorino.it/monitoraggio-acque/>

|  | $\tau_{r}(flow mode)= \frac{V_{reaction chamber}}{Flow rate}= \frac{10 mL}{5500 \frac{mL}{min}}=0.0018 min$ | (S1) |
| --- | --- | --- |
|  | $Total cycles in 10 min=\frac{Loop time}{one cycle time}= \frac{10 min}{0.91 min}=11$ | (S2) |
|  | $\tau_{r} \left( loop \right)= \tau_{r}\left( flow mode \right)\cdot total cycles in 10 min=0.0018 min \cdot11=0.02 min$ | (S3) |

| **Average HCO_3_^-^ content in italian drinking waters** | |
| --- | --- |
| **City** | **HCO_3_^-^ concentration (mg/L)** |
| Turin (this work) | 257 |
| Milan | 149 |
| Rome | 412 |
| Naples | 522 |

**Table S2**. Average HCO3^-^ concentration in italian drinking (tap) waters


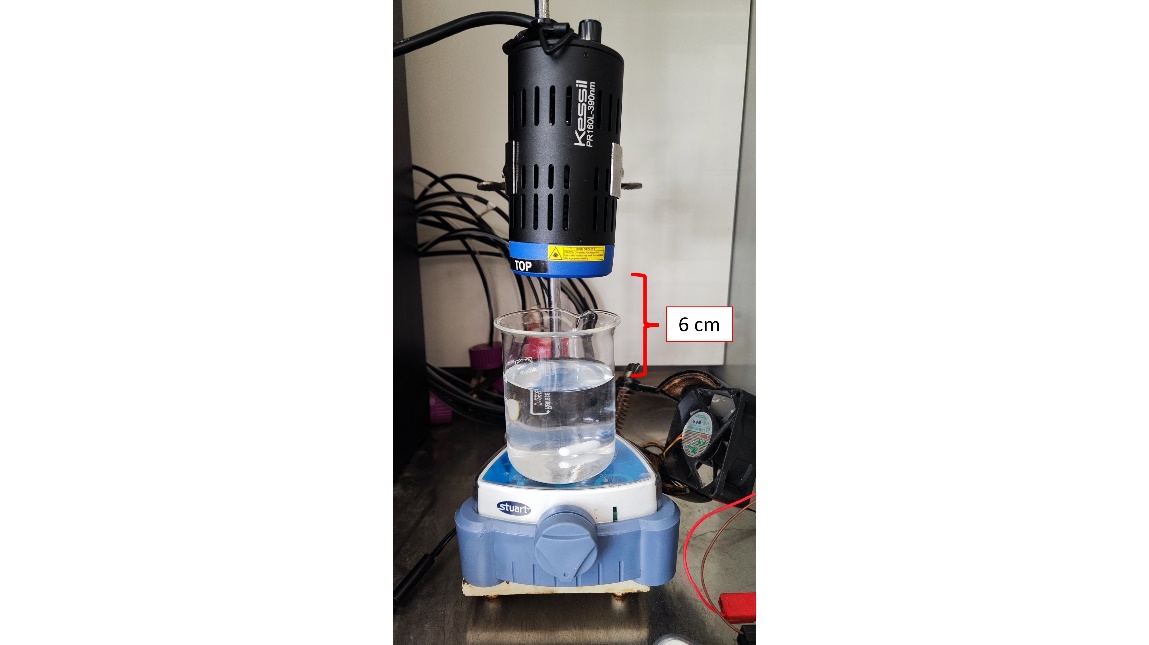


**Figure S2.** Experimental set-up of UV and UV/H_2_O_2_ treatments


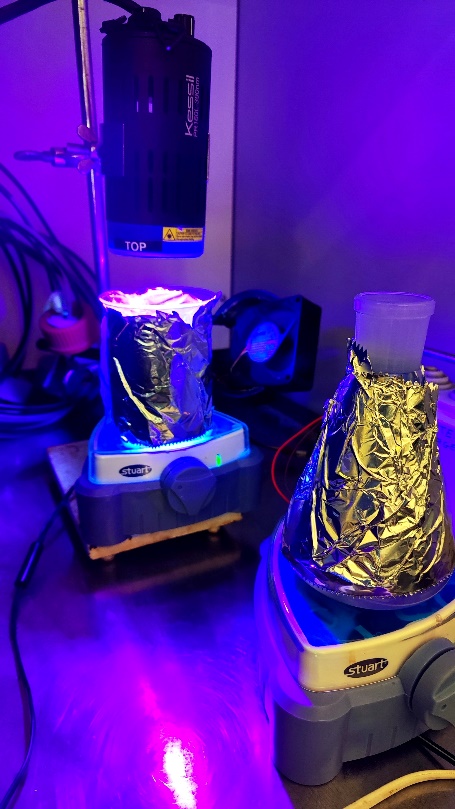


**Figure S3.** Working experimental set-up of UV and UV/H_2_O_2_ treatments


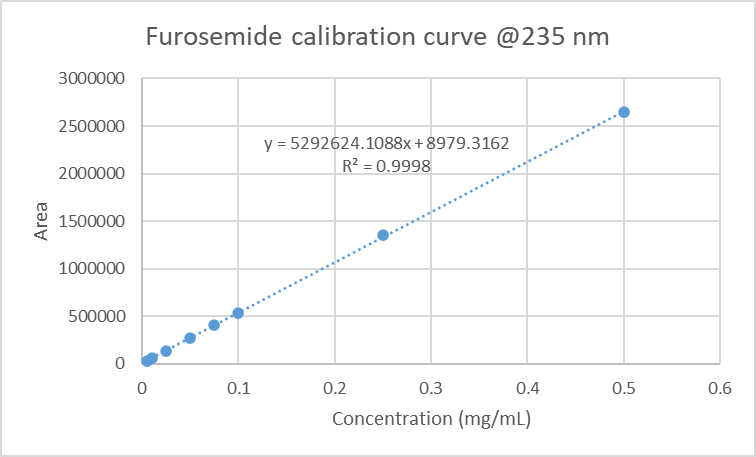


**Figure S4.** Furosemide calibration curve

| **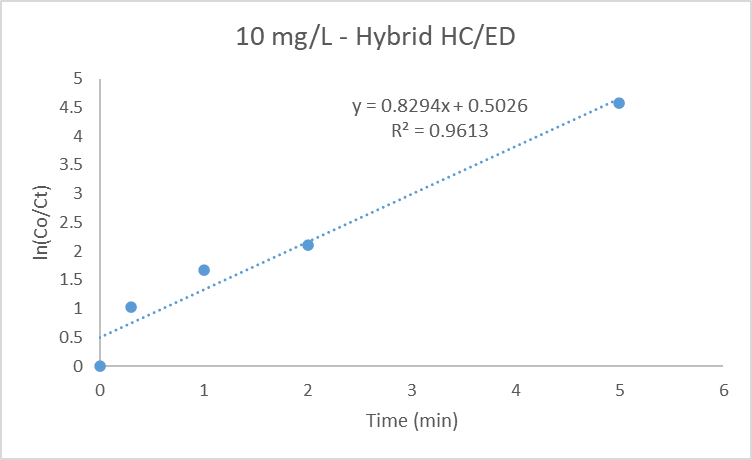** | **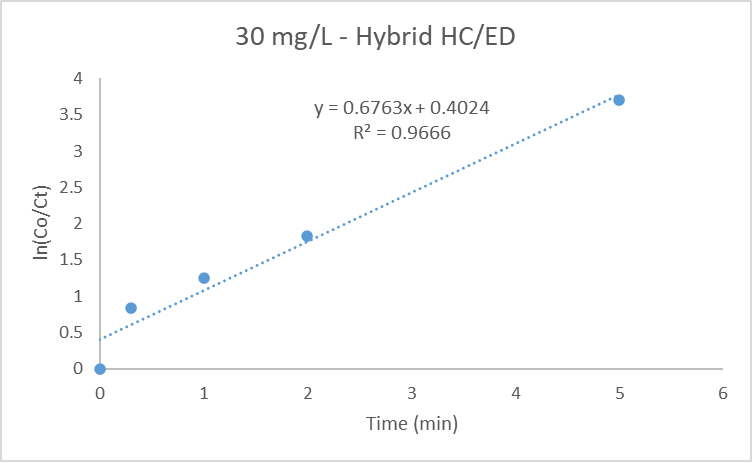** |
| --- | --- |
| **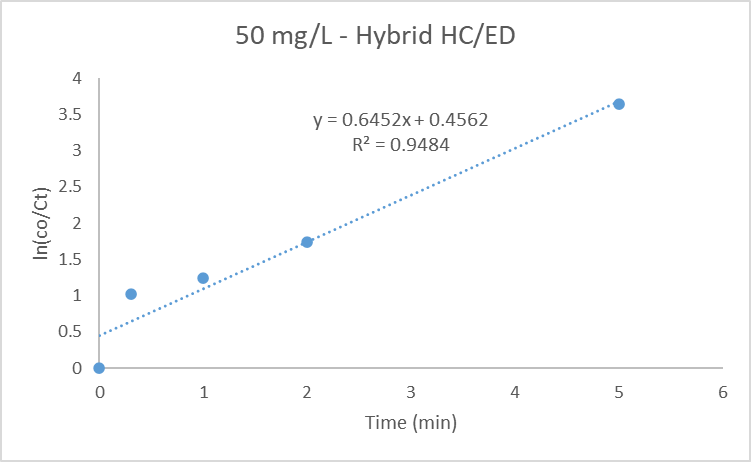** | **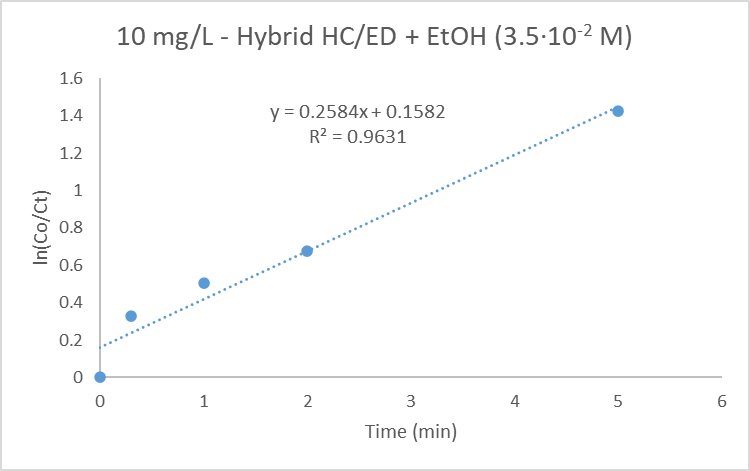** |
| **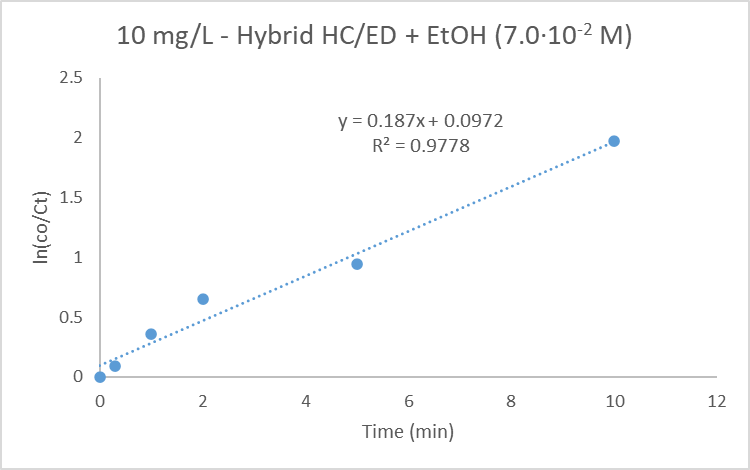** | **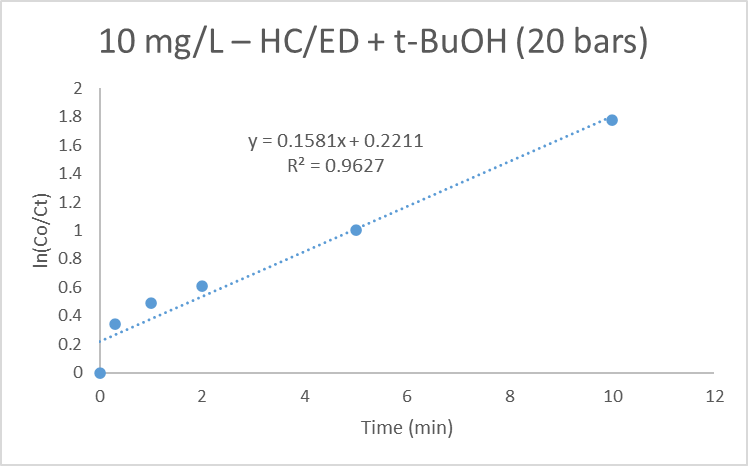** |
| **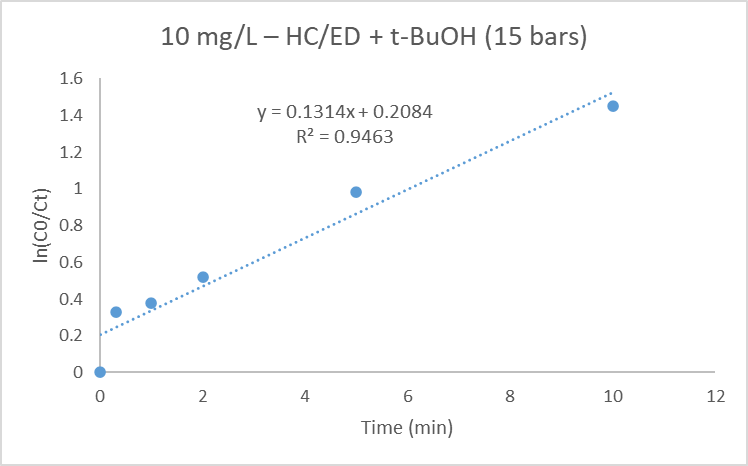** | **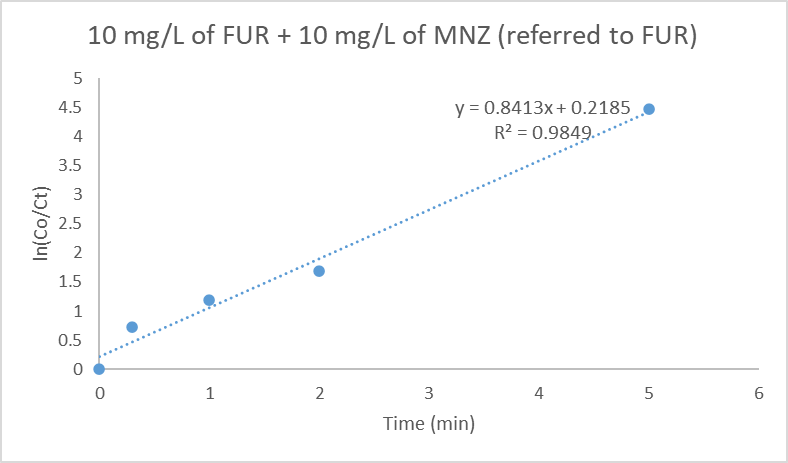** |

**Figure S5.** Linear regression of HC/ED experimental data according to pseudo-first order kinetic model

| **Type of treatment** | **k (min^-1^)** | **R^2^** | **Energy efficiency estimation (mg/kWh)^a^** |
| --- | --- | --- | --- |
| 10 mg/L – Hybrid HC/ED | 0.8294 | 0.9613 | 82 |
| 30 mg/L – Hybrid HC/ED | 0.6763 | 0.9666 | 226 |
| 50 mg/L – Hybrid HC/ED | 0.6452 | 0.9484 | 350 |
| 10 mg/L – HC/ED + EtOH (3.5·10^-2^ M) | 0.2584 | 0.9631 | 74 |
| 10 mg/L – HC/ED + EtOH (7.0·10^-2^ M) | 0.1870 | 0.9778 | 62 |
| 10 mg/L – HC/ED + *t*-BuOH (20 bars) | 0.1581 | 0.9627 | 58 |
| 10 mg/L – HC/ED + *t*-BuOH (15 bars) | 0.1314 | 0.9463 | 55 |
| 10 mg/L of FUR + 10 mg/L of MNZ | 0.8413 (FUR) | 0.9849 (FUR) | - |

^a^10 minutes treatment

**Table S3.** Kinetic constants and energy efficiency values estimated for the performed hybrid HC/ED treatments on furosemide.


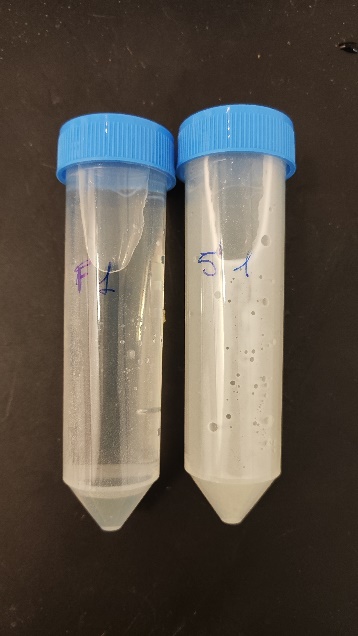

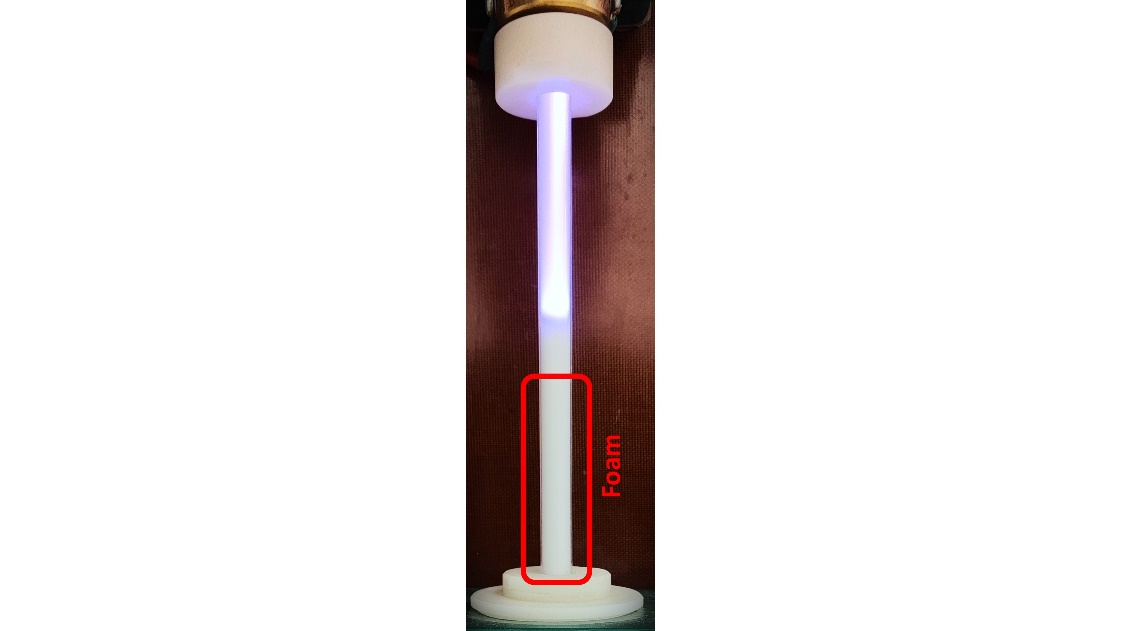

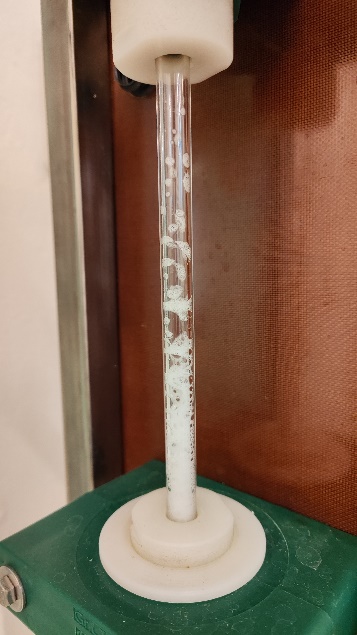


**Figure S6**. a) Foam after 1 minute of treatment in the reaction chamber. b) Foam in reaction chamber at the end of the treatment c) Sample collected after 5 minutes of treatment


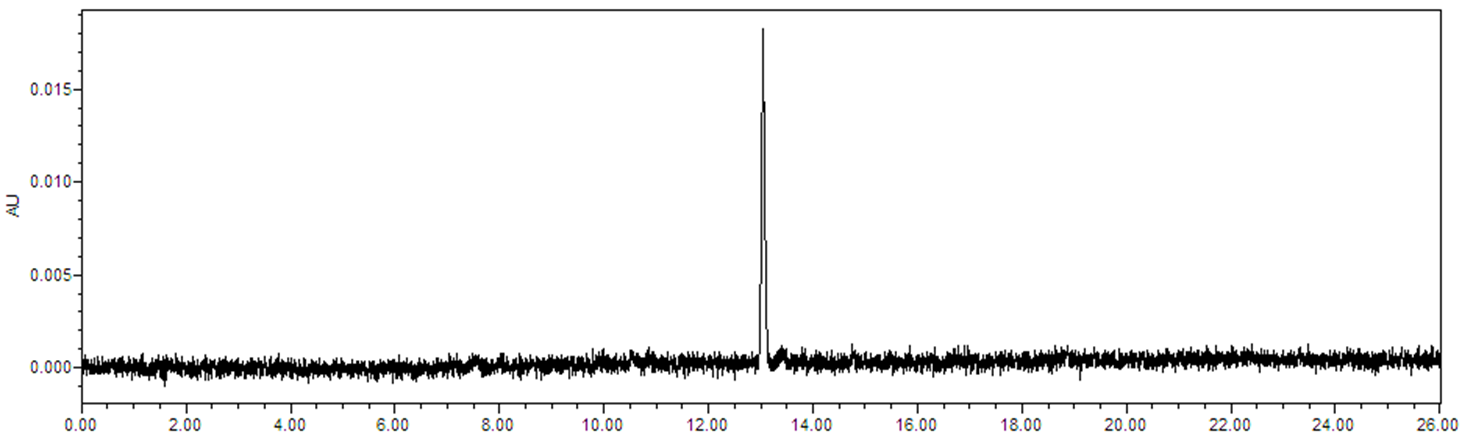


**Figure S7**. HPLC chromatogram extracted at 235 nm of FUR solution (10 mg/L) treated under HC/ED in flow mode (64% of degradation rate). Rt=13.174 🡪 Furosemide


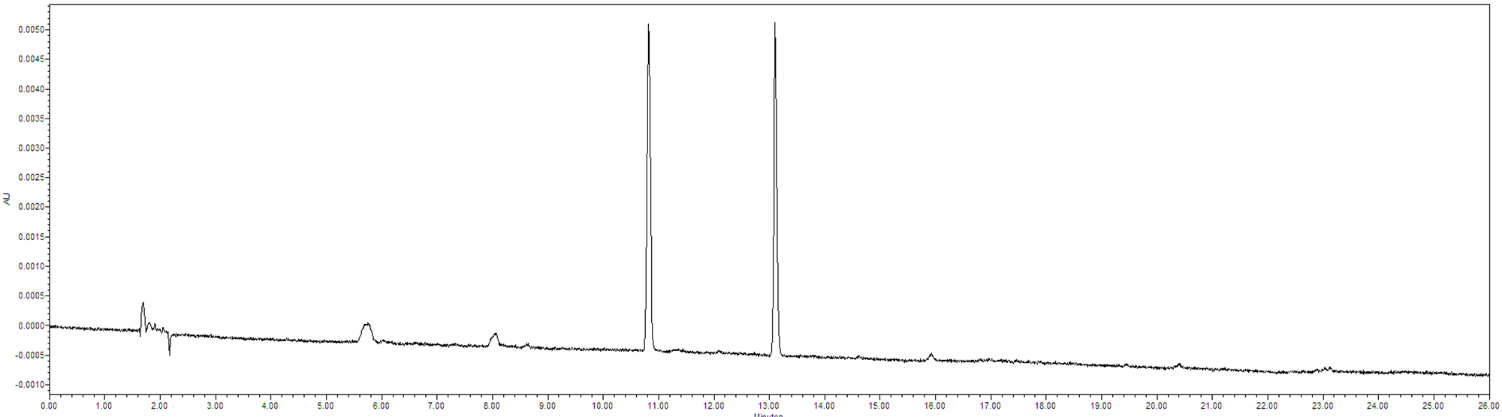


**Figure S8.** HPLC chromatogram extracted at 235 nm of FUR solution (10 mg/L) treated under UV irradiation for 3 hours (88% degradation rate). Rt=13.118 🡪 Furosemide. Rt=10.846 🡪 by-product
